# Mental health and urban living in sub-Saharan Africa: major depressive episodes among the urban poor in Ouagadougou, Burkina Faso

**DOI:** 10.1186/s12963-016-0084-2

**Published:** 2016-05-05

**Authors:** Géraldine Duthé, Clémentine Rossier, Doris Bonnet, Abdramane Bassiahi Soura, Jamaica Corker

**Affiliations:** Institut national d’études démographiques (INED – UMR CEPED) INED, 133 bd Davout, 75020 Paris, France; Institut d’études démographiques et du parcours de vie (I-DEMO), Faculté des Sciences économiques et sociales - Uni Mail, 40, bd Pont d’Arve, CH 1211 Genève 4, Switzerland; Institut de recherche pour le développement (IRD – UMR CEPED) CEPED, 19 rue Jacob, 75006 Paris, France; Institut supérieur des sciences de la population (ISSP), ISSP - Université de Ouagadougou, BP 7118 - 01 Ouagadougou 01, Burkina Faso

**Keywords:** Mental health, Major depressive disorder, Sub-Saharan Africa, Health and Demographic Surveillance System (HDSS), Urban health

## Abstract

**Background:**

In sub-Saharan African cities, the epidemiological transition has shifted a greater proportion of the burden of non-communicable diseases, including mental and behavioral disorder, to the adult population. The burden of major depressive disorder and its social risk factors in the urban sub-Saharan African population are not well understood and estimates vary widely. We conducted a study in Ouagadougou, Burkina Faso, in order to estimate the prevalence of major depressive episodes among adults in this urban setting.

**Methods:**

The Ouagadougou Health and Demographic System Site (HDSS) has followed the inhabitants of five outlying neighborhoods of the city since 2008. In 2010, a representative sample of 2,187 adults (aged 15 and over) from the Ouaga HDSS was interviewed in depth regarding their physical and mental health. Using criteria from the Mini International Neuropsychiatric Interview (MINI), we identified the prevalence of a major depressive episode at the time of the interview among respondents and analyzed its association with demographic, socioeconomic, and health characteristics through a multivariate analysis.

**Results:**

Major depressive episode prevalence was 4.3 % (95 % CI: 3.1–5.5 %) among the survey respondents. We found a strong association between major depressive episode and reported chronic health problems, functional limitations, ethnicity and religion, household food shortages, having been recently a victim of physical violence and regularly drinking alcohol. Results show a U-shaped association of the relationship between major depressive episode and standard of living, with individuals in both the poorest and richest groups most likely to suffer from major depressive disorder than those in the middle. Though, the poorest group remains the most vulnerable one, even when controlling by health characteristics.

**Conclusions:**

Major depressive disorder is a reality for many urban residents in Burkina Faso and likely urbanites throughout sub-Saharan Africa. Countries in the region should incorporate aspects of mental health prevention and treatment as part of overall approaches to improving health among the region's growing urban populations.

## Background

The contribution to the global burden of disease of mental and behavioral disorders is substantial and is estimated to be the first leading cause of years lived with disability (YLDs) in 2010 worldwide [1]. A quarter of individuals will likely present at least one such disorder during their lifetime [2]. The epidemiological literature has demonstrated a high prevalence of these disorders in both developed and developing countries [3, 4]. Like communicable and non-communicable diseases, mental disorders contribute to the poor health of populations, and poor mental health has been cited as a hindrance to efforts underway to achieve the United Nations Millennium Development Goals [5, 6]. To date, mental health is rarely taken into consideration in the public health policies of low-income countries, particularly in sub-Saharan Africa [7–9]. However, as these countries move through the epidemiological transition, the burden of disease shifts from being primarily attributable to communicable diseases and early-life mortality towards a greater contribution to the disease burden from non-communicable diseases (NCDs). Consequently, among an increasing proportion of adults, NCDs, including mental and behavioral disorders, become more prevalent.

Among mental and behavioral disorders worldwide, depressive disorders were the leading cause of YLDs in 2010 [1]. Though they are increasingly common among societies which move away from traditional rural modes of organization into modern and urbanized ones [10], major depressive disorders are not specific to the wealthy and remain prevalent in varied cultural and economic contexts [3, 4, 11]. Because of the substantial impact of major depressive disorders have on YLDs and the widely established methods for measuring major depressive episodes, we choose to focus specifically on measuring and analyzing the prevalence of major depressive disorder in our study.

A major depressive episode indicates the onset of a major depressive disorder. Such an episode is diagnosed on evidence of persistent symptoms affecting a person’s mood, perception, and engagement in daily life and normal activities over a specified 15 day period. It is believed that specific symptoms (e.g., feeling guilty or wishing to commit suicide) may be less prevalent in some cultures than in others [12], though this view is not unanimous [13]. The symptoms used to diagnose a major depressive episode are identifiable in all cultures [14], and diagnostic criteria have been standardized at the international level [15, 16].

The manifestation of major depressive disorder arises from a combination of mechanisms that can be classified into three groups: (i) biological factors, which are relatively poorly identified, (ii) psychological factors involving stressful events (e.g., family break-up or being subjected to physical violence), and (iii) sociodemographic factors (e.g., sex, age, poverty, unfavourable environment) [2]. In low- and middle- income countries, poverty has been cited as underlying factor of mental disorders, including major depressive disorder, and there are believed to be interactions between poverty and depression [2, 4]. Empirical studies have come to inconclusive findings on the relationship between the occurrence of a major depressive disorder and socioeconomic factors, specifically those related to inequality [17, 18]. More consensually, major depressive disorder is consistently associated (in a bidirectional association) with individuals’ overall state of health [6, 19], disabilities [20], risk behaviors such as smoking and alcohol consumption [21, 22], and with mortality [20].

Major depressive disorder prevalence remains largely unknown in many sub-Saharan African countries. Studies carried out to date have been single-country studies, almost exclusively in Anglophone countries, rarely conducted at national level, and have produced a wide range of prevalence estimates. Two recent studies that estimated the 12-month prevalence for major depressive episode found low or moderate rates, including a survey conducted by the World Health Organization (WHO) in Nigeria which found a prevalence of 1 % [7, 23] and a survey in South Africa that estimated a prevalence of 4.9 % [24]. A review of surveys carried out in Ethiopia found a higher range of prevalence, from 7 to 11 %, depending on the measurement tool [25]. Conversely, some studies have found much higher prevalence rates, including some studies dating as far back as the 1960s [26] and a 1997 study estimated more than 30 % of women living in urban areas in Zimbabwe had experienced anxiety or depressive disorders in the past 12 months [27]. The difference in prevalence estimates, measurement tools, and variation in periods of observation means there are no generalizable estimates of the prevalence of major depression disorder throughout the region.

The rapid urbanization which currently characterizes the African continent is associated with changes in socioeconomic and environmental conditions that are theorized to affect people’s mental health, though process has not been sufficiently appreciated [28]. These conditions include frequent migrations and the weakening of social ties, as well as increases in stress and violence levels related to urban living [4, 12, 29, 30]. Moreover, while poor urban residents are on average better-off than rural residents, they may experience a greater sense of social inequity, as they often live side by side with the richest segment of the population that is concentrated in cities [28]. Also, while adults in cities in the global south generally have better health outcomes than their rural counterparts, at a global level they still experience relatively high levels of infectious diseases, premature non-communicable diseases, accidents, and difficulty accessing health care. Despite indications that urbanites in sub-Saharan may have increased risk of experiencing major depressive episodes, the mental health of these urban residents is often overlooked, in part because of a lack of reliable estimates of the prevalence of mental and behavioral disorders. This, in turn, may hamper the establishment services and training of skilled staff to prevent and treat these disorders among the growing urban population.

Our study focuses on Burkina Faso, a landlocked country of 15 million in West Africa. Mental health care in the country is provided in hospital psychiatric wards or the psychiatric care units of medical centers but is grossly inadequate in meeting the population’s needs, even in the urban centers where such services are concentrated [8]. Like many countries in sub-Saharan Africa, Burkina Faso has seen a substantial increase in the proportion of its population that resides in urban areas. While Burkina Faso's population was only 14 % urban in 1990, but it is estimated by 2015 that nearly 30 % of Burkinabe will live in cities [31]. Ouagadougou, the capital, is a sprawling city with over 2 million inhabitants and estimated to be the second-fastest growing city in sub-Saharan Africa [32].

As urbanization continues in Burkina Faso and across sub-Saharan Africa, an ever-increasing proportion of the population will be found in cities, making understanding mental health in these growing urban areas increasingly important for improving health indicators throughout the region. To our knowledge, no previous study on depression has been conducted in Burkina Faso. The information collected in the 2010 Health Survey enabled us to estimate for the first time the proportion of urban adults in Ouagadougou who had experienced a major depressive episode at the time of the study, as well as to identify associations between a major depressive episode and demographic, socioeconomic and health status characteristics.

## Data and methods

This study uses data collected as part of the Ouagadougou Health and Demographic System Site (Ouaga HDSS), which has been monitoring the inhabitants of five neighborhoods located on the periphery of the city since 2008. Two of these neighborhood areas are planned formal neighborhoods, while the other three are informal settlement areas [33]. Residents in the informal settlements are on average poorer than those in the formal areas, are more likely to be migrants who were born in rural areas, and have higher levels of mortality [34]. The protocols for the health survey and routine HDSS data collection were approved by the Ethics Committee for Health Research of the Ministry of Health of Burkina Faso. All participants provided their written informed consent to participate in this study.^1^ All households from the original census were then visited on average every seven months to record births, deaths, changes in marital status, and migrations for all members of the households. As of January 2010, the Ouaga HDSS was following 76,452 residents, 61 % of whom were older than 15 at that time.

In 2010 a specific health survey was conducted on a representative sample of 2,187 individuals among the monitored Ouaga HDSS population in order to collect detailed information about health care, health behaviors, health problems including current major depressive episodes (with alcohol consumptions, they are the only information related to mental and behavioral disorders collected). Prior to the implementation of this survey, interviewers were given a one-week training on the Health Survey questionnaire by a physician and a social researcher. Interviews were face-to-face with the respondents and information was directly inputted on pocket PCs, and this data was managed directly by the Ouaga HDSS team. A multistage stratified sample survey was conducted to select households by grouping people by age groups (0–14, 15–49, 50+, 65+ years old). After selection of one household, all eligible members were interviewed.

Ouagadougou is dominated by the Mossi ethnic group and most inhabitants speak Moore as their first language, although French, the official language, is widely spoken among the educated. The module devoted to mood disorder was translated from French into Moore^2^ and offered to respondents in one of these two languages; as during the initial HDSS census, 62 % of the interviews for the 2010 HDSS Health Survey were conducted in Moore and 35 % in French. Ultimately, 2,195 people over the age of fifteen were interviewed for the 2010 HDSS Health Survey, but eight of them did not answer the specific module because their proficiency in Moore or French was considered insufficient by the interviewer, resulting in a final sample of 2,187 individuals.

To identify major depressive episodes in respondents at the time of the interview, we used the Mini-International Neuropsychiatric Interview (MINI) questionnaire, which was developed using the psychiatric criteria from the fourth edition of the Diagnostic and Statistical Manual of Mental Disorders (DSM-IV) [16, 35]. Using the MINI format, respondents were asked two questions about 1) overall mood deterioration and 2) loss of interest in usual activities. People who said “yes” to at least one of these first two questions were then asked a set of seven additional questions that concerned appetite, sleep, behavior, fatigue, self-esteem, concentration and suicidal thoughts. Following the MINI criteria, a major depressive episode was diagnosed when a respondent said that he or she had consistently experienced at least five symptoms, including at least one of the first two, during the last fifteen days.

We calculated the proportion of respondents experiencing a major depressive episode at the time of the study among the different groups of population. These descriptive analyses were weighted and stratified to take into account the design effect. We built regression models to investigate associations with the risk of being depressed. For the multiple logistic regression analysis, we present three models: the first model includes only demographic characteristics, the second model also includes socioeconomic characteristics and the third model adds in health characteristics which also strongly interact both with demographic and socioeconomic characteristics (for instance, age and chronic diseases). Independent variables were selected to identify characteristics commonly cited as linked to major depressive disorder in the literature [2, 17–19, 36–38].

Data on social, demographic, economic, and health characteristics come from two sources. First, we use the basic sociodemographic information collected as part of the HDSS, which includes data on individuals’ sex, age, ethnicity, religion, marital status, place of birth, education, type of residence (formal or informal neighborhood), and a measurement of household standard of living. For the household's standard of living, we created a proxy variable based on an index that accounts for the presence of durable goods (a refrigerator and a television) and the most expensive mode of transport available in the home. The coefficient attached to each good is derived from a principal components analysis (PCA). Four categories of households were defined. The poorest households do not own any of the listed goods (but could have a bicycle, as most households in Ouagadougou own at least a bicycle), while most of the wealthiest households owned a refrigerator, a television and a motorbike, and half of them have a car. Second, we use more specific data on the same individuals collected from the 2010 HDSS Health Survey. This includes information on reported chronic diseases, obtained by asking the question “Has a doctor or nurse ever told you that you have/suffer from…?” and listing a series of common diseases and health problems (hypertension, heart attack, coronary heart disease, angina, congestive heart failure or another heart problem, cerebral embolism, cerebrovascular accident, stroke or thrombosis, chronic lung diseases such as chronic bronchitis, emphysema and asthma, stomach ulcer, and other diseases). Data was also collected on whether respondents had any accidents during the last 12 months that required treatment, physical violence suffered in the last 12 months, moderate or severe functional limitations (seeing, hearing, walking, washing oneself, getting dressed, communicating), alcohol consumption, smoking, and any household food shortages during the past month.

## Results and discussion

One respondent in ten reported feeling either “depressed, sad or empty” or to have “lost interest or enjoyment in things that they used to enjoy” in the fifteen days prior to the survey. Among this group, 65.7 % reported having trouble sleeping; nearly half experienced a noticeable change in appetite or weight, or felt tired; 35.2 % reported a change in behavior (moving slowly or feeling particularly agitated); 25.9 % a lack of self-esteem; 15.6 % suicidal thoughts; and 12 % had difficulty concentrating. Ultimately, the proportion of people aged fifteen and older who were going through a major depressive episode at the time of the study was estimated to be 4.3 % (95 % CI: 3.1–5.5 %) (Table 1).

**Table 1:** Major depressive episodes at the time of survey among adults, Ouaga HDSS 2010 Health Survey

Proportion of people who said they consistently had the following symptoms for at least during the last 15 days	% (_95%_ CI)
Among all respondents (*N* = 2,187)	
Depressed, sad or empty	9.0 (7.2–10.8)
Loss of interest or enjoyment	7.2 (5.5–8.9)
One or both	9.9 (8.1–11.8)
Among respondents who said they had at least one of the first signs (*N* = 212.5)	
Unintentional changes in appetite or weight	46.0 (37.6–54.3)
Trouble sleeping	65.7 (57.5–74.0)
Moving more slowly, or feeling agitated	35.2 (27.0–43.3)
Tired, lacking energy	43.6 (35.1–52.0)
Feeling worthless or guilty	25.9 (18.6–33.2)
Difficulty thinking, concentrating or making decisions	12.0 (6.7–17.2)
Suicidal thoughts	15.6 (9.6–21.5)
Proportion of people experiencing a major depressive episode (*N* = 2,187)	4.3 (3.1–5.5)

Notes: Adults are respondents aged 15 years and older

The odds of experiencing a major depressive episode were associated with a number of demographic and socioeconomic variables, as well as with poorer health status. A greater proportion of women were diagnosed with a major depressive episode than men (4.9 % vs 3.7 %), but this difference is small and not significant in the multivariate analysis (Table 2). There were some significant differences according to age, which showed a bell shaped association with major depressive episode, with the highest risk among the middle-age groups (30–49 years), although the significance disappears after controlling for socioeconomic and other health variables which interact with age in the third model. Among the sociodemographic and cultural characteristics, ethnicity and religion show significant associations with a major depressive episode: OR 2.2 (*p* < 0.05) for non-Mossi compared with Mossi, OR 0.5 (*p* < 0.05) for Christians compared with Muslims.

**Table 2:** Proportion reporting and odds ratios of a major depressive episode at the time of the survey by different characteristics, Ouaga HDSS 2010 Health Survey

	Freq^(a)^	% classified as depressed (95 % CI)	Model 1	Model 2	Model 3
			OR	OR	OR
Total survey population	2,187	4.3 (3.1–5.5)			
Male	982	3.7 (2.3–5.1)	1.0	1.0	1.0
Female	1,205	4.9 (3.3–6.4)	1.3 (0.9–2.1)	1.3 (0.8–2.2)	1.5 (0.8–2.7)
15–19 years old	424	1.6 (0.0–3.1)	1.0	1.0	1.0
20–29 years old	880	4.0 (2.3–5.7)	2.7 (0.9–7.8)	2.6 (0.8–8.4)	1.6 (0.5–5.6)
30–39 years old	519	6.3 (3.5–9.0)	4.3 (1.5–12.5)**	4.4 (1.3–15.5)*	2.2 (0.6–8.4)
40–49 years old	266	5.8 (2.1–9.5)	4 (1.3–12.9)*	3.9 (1.0–15.3)*	2.3 (0.5–9.8)
50 and older	98	4.5 (2.4–6.5)	2.0 (0.7–5.9)	2.1 (0.6–7.9)	0.9 (0.2–3.6)
Mossi (majority) ethnic group	1,991	4.0 (2.8–5.2)		1.0	1.0
Any other ethnic group	196	7.7 (2.5–12.8)		2.2 (1.1–4.2)*	2.2 (1.0–4.5)*
Muslim	1,215	5.2 (3.5–6.8)		1.0	1.0
Christian	849	3.7 (1.9–5.5)		0.8 (0.5–1.2)	0.5 (0.3–0.9)*
Other	123	0.3 (0.0–0.7)		1.2 (0.2–7.8)	1.6 (0.2–12.1)
Single	801	3.4 (1.5–5.3)		1.0	1.0
Married	1,309	4.8 (3.2–6.3)		0.7 (0.3–1.5)	0.7 (0.3–1.6)
Widowed or divorced	77	6.1 (0.8–11.3)		0.6 (0.2–1.8)	0.6 (0.2–1.9)
Born in Ouagadougou	660	5.1 (2.8–7.3)		1.0	1.0
Born in another urban area	127	5.1 (0.0–10.7)		0.9 (0.4–2.1)	1.2 (0.5–3.1)
Born in a rural area	990	4.5 (2.8–6.2)		0.6 (0.3–0.9)*	0.7 (0.4–1.2)
Born in another country	294	3.3 (0.7–6.0)		0.5 (0.2–1.2)	0.7 (0.3–1.8)
Place of birth unknown	116	0.2 (0.0–0.6)		0.2 (0.0–2.4)	0.2 (0.0–2.1)
Currently at school	348	1.9 (0.0–3.8)		0.6 (0.2–2.0)	0.8 (0.2–3.1)
Attended school	792	4.4 (2.5–6.3)		1.0	1.0
Did not attend school	896	5.2 (3.3–7.0)		1.2 (0.7–2.2)	1.4 (0.7–2.6)
Education unknown	151	4.3 (0.2–8.4)		1.3 (0.4–4.0)	1.2 (0.3–4.0)
Neighborhood: planned	1,129	3.7 (2.1–5.2)		1.0	1.0
Neighborhood: unplanned	1,058	5.0 (3.1–6.8)		1.2 (0.7–2.1)	1.1 (0.6–2.0)
Standard of living – poorest	410	7.2 (3.7–10.7)		2.7 (1.2–6)*	2.7 (1.2–6.2)*
Standard of living – poorer middle	446	1.9 (0.1–3.6)		1.0	1.0
Standard of living – wealthier middle	573	4.1 (2.0–6.2)		1.7 (0.8–3.8)	1.5 (0.7–3.6)
Standard of living – wealthiest	758	4.4 (2.2–6.5)		2.5 (1.1–5.5)*	2.0 (0.9–4.8)
HH had no/few food shortages	1,892	3.5 (2.3–4.6)		1.0	1.0
HH had frequent food shortages	284	10.2 (5.4–15.0)		2.4 (1.5–4.0)***	1.8 (1.0–3.1)**
No chronic disease reported	1,729	1.8 (1.0–2.6)			1.0
One chronic disease reported	323	8.5 (4.4–12.5)			2.2 (1.2–4.0)***
Two or more chronic diseases	135	26.5 (17.5–35.5)			6.7 (3.5–12.7)***
No accident reported	1,990	4.0 (2.8–5.3)			1.0
Accident reported (past 12 mo.)	197	7.2 (2.3–12.0)			1.9 (0.9–4.0)
No physical violence reported	2,156	4.2 (3.0–5.3)			1.0
Suffered violence (past 12 mo.)	31	16.1 (0.0–35.2)			4.7 (1.2–19.1)*
No functional limitation reported	1,808	2.3 (1.4–3.2)			1.0
Only slight limitations	244	8.6 (4.3–12.9)			1.7 (0.9–3.2)
At least one severe limitation	135	23.4 (14.9–31.8)			4.3 (2.2–8.2)***
Does not drink alcohol	1,535	4.1 (2.8–5.3)			1.0
Drinks once a week or less	482	3.5 (1.4–5.6)			1.7 (0.8–3.6)
Drinks more than once a week	168	9.1 (4.1–14.0)			2.4 (1.1–5.5)*
Occasional smoker or non-smoker	1,994	4.0 (2.8–5.1)			1.0
Regular smoker	192	8.1 (3.3–12.8)			1.6 (0.6–3.8)
−2 Log likelihood			717.148	679.749	565.699

Statistical significance: *** *p* < 0.001, **p < 0.01, * *p* < 0.05

Notes: *HH* household

(a) Weighted frequencies

Notably, migrants (defined as those born in rural areas) were less likely to suffer from a major depressive episode, though this association of type of residence at birth disappears once all other health characteristics are controlled for. Experiencing a major depressive episode was significantly associated (*p* < 0.05) with standard of living. Both the poorest and the wealthiest were more likely to be experiencing a major depressive episode at the time of the survey (OR 2.7 for the poorest and 2.5 for the wealthiest) compared to the poorer middle category. The latter has the lowest rate of major depressive episodes and no significant difference from the wealthier middle category in the full model. However, only the poorest have a significant higher risk in the model 3 which include health characteristics. Individuals who live in households with frequent food shortages in the previous month also have a higher odds of being depressed (OR 1.8, *p* < 0.01) than those who did not report food shortages. Physical health is strongly associated with reporting a major depressive episode: people who reported one chronic disease have much higher risks of being identified as depressed (OR 2.2, *p* < 0.001), and the risk is even much higher among those who reported having at least two chronic diseases (OR 6.7, *p* < 0.001). We also found a strong association of functional limitations and experiencing a major depressive episode (OR 4.3 for severe ones, *p* < 0.001). People who reported having being victim of a physical violence in the past year had higher odds of being depressed than those who had not (OR 4.7, *p* < 0.05). Last, people who drink regularly (more than once a week) had higher odds of being depressed than those who don’t drink (OR 2.4, *p* < 0.05).

Previous studies on depression in sub-Saharan Africa found substantial variation in prevalence levels, depending on the study location, and most cited measure the prevalence of episodes in the last 12 months or over the entire life time [4, 20, 24, 27]. Our study found that 4.3 % (95 % CI: 3.1–5.5 %) of individuals in an urban setting over the age of fifteen were experiencing a major depressive episode. This estimate is not negligible, particularly considering our analysis limited the measurement of major depressive episodes to those diagnosed at the time of the survey, suggesting an even greater prevalence of the population who experience a major depressive episode over their lifetime.

Our results confirm a number of broad findings on the associations with depression from previous studies, including those carried out in sub-Saharan Africa. In our study population, major depressive episodes were strongly associated with self-reported chronic diseases and disability, underscoring the well-established link between depression and other health problems (chronic diseases [19] and physical limitations [20]). Several mechanisms explain this significant co-morbidity: on one hand, being sick is a risk factor of a major depressive disorder, on the other hand, having a major depressive disorder may lead to the onset of diseases, hasten their development or even interfere with their treatment [36]. In addition, access to medical care remains limited in sub-Saharan Africa, even in urban areas. Treatment is primarily geared towards communicable diseases and maternal and child health, despite the growing adult population that is more susceptible to chronic conditions and functional problems related to ageing and illness. In Burkina Faso, not only is prevention practically nonexistent for such health problems, but available care for chronic diseases is also extremely rare or simply financially inaccessible because people do not have health insurance. In such conditions, when people learn they have a chronic disease or disability and their health state deteriorates, they may lose sight of the possibility of an improvement in their condition, which could explain a greater propensity for depression. This association may be reinforced by such kind of study based on self-reported information. Women are generally more likely to experience depression than men, although the degree of the gender gap varies depending on the context and the content of gendered social roles [36–38]. In our study population, this gender gap is not maintained when holding all other covariates constant. This is not the only study where such result has been found [39], although in both studies (this one and the one quoted) the prevalence was higher among women than men and the absence of significance may be simply due to a methodological issue linked to small sample sizes. Regarding marital status, prevalence of major depressive disorder is generally lower among married people than other single people, especially those who are widowed or divorced [40, 41]. In this survey, only a few persons were widowed or divorced which probably explain the absence of significance. Having been a victim of physical violence, another known correlate of depression [4, 12, 29], was also found to be significantly associated with a major depressive episode in Ouagadougou, as was living in a household that experienced frequent food shortages. Regarding unhealthy behaviors, smokers and alcohol drinkers were more likely to report symptoms of a major depressive episode (though this could also be a factor of reverse causation [36]), in line with another general finding from the broader literature on major depressive disorder, but an association is only found for drinkers, and not for smokers. Last, our results showed that the middle-aged population is the most likely to experience a major depressive episode, this confirms findings from most previous studies [36] but in Ouagadougou, socioeconomic and health characteristics explain this association.

The findings here related to the association of sociocultural differences and major depressive disorders are more likely to be dependent on the Ouagadougou context and may not be generalizable. For example, the relationship of ethnicity with a major depressive episode is difficult to interpret, particularly as the non-Mossi group in Ouagadougou is small (only 9 % of the population of the Ouaga HDSS survey population) and heterogeneous. While religion has been generally found to generally provide social support and prevents depression, there is little research specifically on Christian/Muslim differentials with the exception of studies focusing on religion as a minority status carried out in some developed country settings. We found that in Ouagadougou, after controlling for alcohol consumption (which largely implicates only Christians), Muslims are more likely to experience a major depressive episode than Christians, although this could be a reflection of influences associated with religion in this specific context (e.g., minority status or cultural differences, and while it is beyond the scope of this project to qualify this difference, future qualitative research could explore this result). Finally, while a link between migration and mental health is often cited in the global literature [12], empirical results do not always show a systematic relation, particularly in the case of internal migratory flows from the countryside to the cities [42]. In Ouagadougou, having been born in a rural area (and, hence, having migrated to Ouagadougou) was associated with fewer episodes of a major depressive disorder, but this association disappeared after controlling for other measures of health status. Although education is generally associated with a lower likelihood of developing depression [17], we found no significant relationship between education and depression in our survey sample.

Previous empirical studies in other settings and much of the theory on depression emphasize the association between depressive disorder and poverty. Our results confirm that the poorest (characterized by the standard of living and the experience of food shortages) are more likely to experience a major depressive episode [4, 17], and this is supported by findings from a previous qualitative research project on poverty and psychological vulnerability conducted in the Ouaga HDSS setting [43]. Though, we found an unexpected non-linear association between major depressive episodes and standard of living among individuals in Ouagadougou. The association between reporting symptoms of major depressive episode and standard of living in our study population follows a U-shaped pattern: it is highest among the poorest and the wealthiest, and lowest among those in the middle, though the association is stronger among the poorest than the wealthiest for which the significance disappears once controlling the health characteristics. The standard of living is here measured based on household’s material goods, and does not necessarily reflect the household’s financial situation at the time of the study, but rather its capacity to save or invest in past years. This cross-sectional indicator cannot measure an individual's satisfaction with his or her life trajectory, known to be associated with depression [44], as an individual's satisfaction with his or her current degree of wealth often depends on one's previous level of wealth. It is possible that some of those considered wealthy here have in fact experienced a recent decline in their standard of living, living in the periphery of Ouagadougou. Another hypothesis could be that the richest individuals might be leading more modern and individualistic lifestyles, which are believed to be more conducive to the development of major depressive disorder than traditional ones [10]. Alternatively, living in an informal neighborhood (which has higher rates of poverty, generally lacks infrastructure, and is further from health services) could be assumed to be positively associated with major depressive episodes. We did not find, however, that neighborhood type was significantly correlated with the likelihood of experiencing a major depressive episode in this Ouagadougou setting.

This study has several limitations that are worth noting. One is related to the self-reporting of information, which could be biased as social factors impact how one person experiences and expresses one’s health state. This potential bias holds true for self-rated health in general, but self-reported health has general been shown to be a good indicator of objective health [45]. Moreover, the identification of a major depressive episode is not a direct answer given by the respondent, but rather a condition identified based on a conditioned series of simultaneous symptoms. However, some potential biases remain: first, though the MINI tool is an international standard [15, 16], it has been developed in Western countries and may not be sensitive to all presentations of major depressive episodes in Ouagadougou; second, some of the questions asked may seem unusual or intrusive to respondents which may encourage them to answer in the negative; third, it is always possible that interviewers may ask prompt questions about overall mood deterioration and loss of interest in usual activities in such a way that the respondent will answer negatively allowing them to avoid the filtered questions and finish the interview more quickly. Taking these factors into account, the estimated proportion of current major depressive episode found here could be underestimated and is perhaps best considered as a minimum prevalence estimate. Additionally, results from multivariate analysis are based on regression models, the associations highlighted by our analysis depend on the specific set of variables introduced in the model and we have potential unmeasured confounders. Due to small frequencies, we couldn’t assess the existence of a clustering effect of the household level, which could be present as in some cases multiple members of the same household were interviewed. For the same reason, we did not run separate models for each sex, as certain associations are probably stronger for males (e.g., alcohol consumption), while some are for females (e.g., marital dissolution). Finally, the significant associations found in this study cannot necessarily be interpreted as causal factors for a major depressive disorder. Further research, more specifically devoted to mental health with a larger sample size, is required to confirm these first results.

## Conclusions

Our study found that 4.3 % (95 % CI: 3.1–5.5 %) of residents in Ouagadougou were currently suffering from a major depressive episode at the time of the survey, with an overall risk of a lifetime major depressive disorder among this population likely higher. We found that experiencing a major depressive episode in Ouagadougou was associated with a reported chronic disease, disability, household food shortages, having experienced physical violence, and drinking regularly alcohol. Migrant status, neighborhood type (formal or informal), age group, marital status, and gender were not significantly associated with major depressive episodes. A surprising finding from this study showed that, even if the poorest were most susceptible to major depressive disorder, the wealthiest segments of society also tend to be more vulnerable to depression in this setting.

Although urban populations in developing countries, like those living in Ouagadougou, still face a heavy burden of disease in the form of infectious diseases, chronic diseases and accidents [34], many individuals suffering from these health problems often also experience from major depressive disorder. This may be particularly true for chronic diseases, which showed a strong association with experiencing a major depressive disorder. As urban populations throughout sub-Saharan Africa continue to grow and experience an upward shift in their age structures, it is likely that an increasing proportion of urban dwellers will experience a major depressive episode in their lifetime, with detrimental effects for their well-being. It is thus critical that, following recommendation from the WHO [46], efforts to improve overall health in urban populations strive to incorporate mental health diagnosis and treatment in a more systematic way within established health systems.

## Footnotes

1 This study included both literate and illiterate respondents. Illiterate respondents were asked to provide a literate witness and the informed consent form was read to both the respondent and the witness. If the respondent agreed to participate, he/she was asked for a finger print on the consent form, and the witness was asked to provide his/her signature on the form as well. The proportion of illiterate respondents here is believed to be similar to that of Ouagadougou as a whole based on a data comparison with the 2010 Burkina Faso Demographic and Health Survey.

2 The Burkinabe Institute for Literacy (*Direction de la Recherche, des Innovations en Éducation Non Formelle et en Alphabétisation, DRINA*)was in charge of the translation of the questionnaire. To ensure accurate translation, a backward translation from Moore to French was also done.
